# Mesoporous Spherical Silica Filler Prepared from Coal Gasification Fine Slag for Styrene Butadiene Rubber Reinforcement and Promoting Vulcanization

**DOI:** 10.3390/polym14204427

**Published:** 2022-10-20

**Authors:** You Xu, Weidong Ai, Jing Zuo, Wentong Yang, Cundi Wei, Shaonan Xu

**Affiliations:** 1 College of Materials Science and Engineering, Jilin University, Changchun 130025, China; 2 College of Material Science and Engineering, Jilin Jianzhu University, Changchun 130118, China

**Keywords:** styrene butadiene rubber composite, sustainability, mechanical properties, promoting vulcanization, coal gasification fine slag

## Abstract

Coal gasification fine slag (CFS) is a solid contaminant produced by an entrained flow gasifier, which pollutes fields and the air in the long term. CFS is a potential polymer reinforcement filler and has been used in polypropylene and acrylonitrile butadiene styrene resins. Coal gasification fine slag mesoporous silica (FS-SiO_2_) was prepared by acid leaching, calcination, and pH adjustment, with a larger specific surface area and less surface hydroxyl compared to the commercial precipitated silica (P-silica). The cure characteristics, crosslink density, mechanical properties, the morphology of the tensile fractures, dynamic mechanics, and rubber processing of the prepared styrene butadiene rubber (SBR) composites filled with P-silica and FS-SiO_2_ were analyzed, respectively. The results indicated that FS-SiO_2_ was dispersed more uniformly in the SBR matrix than P-silica owing to its smaller amount of surface hydroxyl and spherical structure, resulting in a better mechanical performance and wet skid resistance. In particular, the SBR composites with a filler pH of 6.3 exhibited the highest crosslink density and tensile strength, being superior to commercial P-silica. Significantly, the curing time decreased with the increase in the pH of FS-SiO_2_, which caused the rubber processing to be more efficient. This strategy can reduce the cost of rubber composites and the environmental pollution caused by CFS.

## 1. Introduction

Coal gasification slag, consisting of coarse slag (CCS) and fine slag (CFS), is a by-product of entrained-flow coal gasification. Currently, the annual emission of coal gasification slag reaches hundreds of millions of tons [1]. Additionally, the CFS is mainly accumulated and landfilled in slag dumps due to the lack of effective disposal methods [2]. Therefore, heavy metals in CFS, such as Cr, Mn, Ni, Ba, and Zn, not only seriously pollute the soil and the underground water but also pose a high risk to human health throughout the food chain [3]. In addition, the nanometric particles of CFS easily spread into the air and are inevitably inhaled into the lungs by workers and nearby residents, which may increase the risk of respiratory infection and be harmful to the immune system [4]. The coal is burned in the gasifier, and the fly ash flows along the inner wall of the furnace cavity to the bottom, cooling by water before it is collected by the lock bucket, yielding the coal gasification coarse slag. The remaining inorganic molten phase and unburned carbon leave the combustion chamber with the synthesis of the gas, condensed by water vapor. The mixture of particles and water pass through a filter press to create a filter cake. The filter cake is dried and crushed to obtain coal gasification fine slag [5]. In particular, CFS is composed of unburned carbon and inorganic regular microspheres. The composition of the inorganic microspheres in the fine slag mainly includes SiO_2_, containing the oxides of metal elements such as aluminum, iron, and calcium, and the unburned carbon remains loose and flocculent [6,7,8]. The increasing discharge of solid waste CFS and its negative impact on the environment must be given widespread attention. Therefore, the new and high-value-added applications of this solid waste are efficient ways to prevent environmental pollution. Based on the characteristics of coal gasification slag, different functions have been assigned to it by many researchers. Mesoporous silica [9] material, residual carbon adsorbent [10], and Y-type zeolite/carbon porous composites [11] were prepared by acid leaching, floating, and the hydrothermal synthesis method from CFS to absorb the organic pollutants in water. The maximum adsorption capacity of these materials was excellent, benefiting from their large specific surface area, high porosity, and chemical stability [12]. The utilization of CFS in this field achieved the goal of treating waste with waste. Furthermore, high-performance absorbents for CO_2_ capture [13], microwave absorbers [14], conductive powders [15], and air cathodes for zinc–air batteries [16] were prepared from CFS due to the advantages of their hierarchical porous and thermal stability. The utilization of CFS in the abovementioned fields has achieved encouraging results. However, with the increasing emission of CFS, the consumption of CFS in these products cannot meet the requirements. Hence, finding a high-value-added and high-consuming utilization of CFS is of great significance.

Polymers such as rubbers and plastics are widely used in daily life and industrial production. Generally, choosing appropriate fillers not only reduces the costs of production but also reinforces the composites. Currently, carbon black (CB), precipitated silica (P-silica), calcium carbonate, kaolin, etc., are the most widely used fillers for reinforcing rubbers and plastics. Tens of millions of tons of CB and P-silica are consumed every year, which has led to a high consumption of energy and serious environmental pollution. Therefore, using a low-cost and environmentally friendly filler to replace CB and P-silica accelerates the achievement of “carbon neutrality”.

Researchers have attempted to use coal-based solid waste as a substitute for CB or P-silica so as to strengthen rubber matrix. They have mainly focused on fly ash [17], low-rank coal slime [18], and coal gangue [19]. Sombatsompop used raw silica from fly ash as a replacement for P-silica to reinforce natural rubber (NR) and styrene butadiene rubber (SBR), resulting in the decline of the material’s mechanical properties [20]. After being treated with 2 wt% bis (3-triethoxysilylpropyl) tetrasulfide (TESPT), the modified silica from fly ash showed an optimal mechanical performance and could be used to replace the P-silica [21]. Fly ash particles were treated with a silane coupling agent to promote their reinforcement efficiency for polybutadiene rubber and solution styrene butadiene rubber/butadiene rubber by Nabil [22] and Mahmood [23], respectively. In addition, sorbic acid (SA) and tannic acid (TA) were introduced onto the surface of fly ash to improve the filler–rubber interaction and the mechanical properties of rubber composites. Nonetheless, the complex processing of the grafted fly ash and the toxicity of the silane coupling agents limited their industrial production. As a matter of fact, the higher crystalline degree resulted in a lower surface activity of the fly ash, and the smooth surface limited its compatibility with the rubber matrix.

After the acid leaching and calcination process, FS-SiO_2_ had the characteristics of a low crystalline degree, high surface activity, abundant pore structure, larger specific area, and a rough surface [9], which gave it the potential to be used as a reinforcement filler. Zhang found that fine slag silica not only strengthens the polymers but also shows an excellent performance in the removal of volatile organic compounds (VOCs) [24,25]. Additionally, Ai [26] verified the feasibility of CFS as a replacement for calcium carbonate to reinforce styrene butadiene rubber. However, few studies have explored the possibility of replacing P-silica with CFS as a rubber filler. Similar to precipitated silica, surface hydroxyl was shown to exist on FS-SiO_2_ and absorbed the active curing agent, which slowed down the speed of the vulcanization of the rubber. In response to this problem, sodium hydroxide solution was used to adjust the surface hydroxyl amount of FS-SiO_2_ in this study, and the effects of the amount of surface hydroxyl of FS-SiO_2_ on the reinforcing properties and processing performance of styrene butadiene rubber were studied. Considering the ubiquity of rubber products and the possibility of consuming a large amount of CFS, we chose the most widely used styrene butadiene rubber (SBR) [27] as the matrix.

In this study, mesoporous spherical silica fillers of different pH values were prepared from CFS by acid leaching, calcination, and pH adjustment, and their characteristics, such as the surface hydroxyl, particle size distribution, specific surface area, and pore structure, were investigated in detail. The effects of P-silica and FS-SiO_2_ on the cure characteristics of the SBR composites were discussed. The reinforcement efficiency of the mesoporous spherical silica fillers was determined through the analysis of the mechanical properties, crosslink density, morphology of the fracture surfaces, and dynamic mechanical properties of the SBR composites.

## 2. Materials and Method

### 2.1. Materials

The CFS was provided by the Inner Mongolia Yi Tai Group Co., Ltd. (Ordos, Inner Mongolia Autonomous Region, China). The CFS was air-sieved to obtain a finer slag. SBR-1502 was obtained from the Jilin Chemical Industrial Limited Company of China (Jilin City, China). Precipitated silica (P-Silica) with a specific area of 96 m^2^/g was obtained from Jinan Kasong Chemical Co., Ltd. (Jinan City, China) and dried in a vacuum oven overnight at 100 °C before use. The antioxidant 2246, zinc oxide (ZnO), stearic acid (SA), accelerator N-cyclo-hexylbenzothiazole-2-sulphenamide (CZ), 2, 2′-dibenzothiazyl disulfide (DM), and insoluble sulfur (S) were all industrial-grade products, obtained from Kemai Chemical Co., Ltd. (Tianjin City, China). The sodium hydroxide (NaOH), hydrochloric acid (HCl), and toluene were analytical reagents and used as received, obtained from Shanghai Maclean Biochemical Technology Co., Ltd. (Shanghai City, China).

### 2.2. Preparation of the FS-SiO_2_

A total of 30 g CFS was dissolved in 500 mL of 16% hydrochloric acid and stirred at 75 °C for 4 h. According to Zhang’s research [28], 16 wt% HCl was chosen. Then, it was washed 3 times and dried to obtain a powder material. In order to obtain a light-colored filler, the powdered material was burnt in a muffle furnace at 600 °C for 3.5 h [9,24] to remove unburned carbon, and the FS-SiO_2_ was obtained. Finally, the FS-SiO_2_ was dissolved in 350 mL of water, and 0.025 mol/L of sodium hydroxide solution was used to adjust the pH of the mixture to 6.3, 7.3, 8.3, and 9.3, respectively. The initial pH of FS-SiO_2_ was 5.3. We named the FS-SiO_2_ fillers with different pH values as FS-X, where X represents the respective pH value of the FS-SiO_2_ filler.

### 2.3. Preparation of the SBR Composites

Compounds filled with P-silica and FS-X were prepared using a two-roll open mill. X represents the pH value of the slurry of the fillers. First, SBR-1502 (100 phr) was masticated using an open two-roll mill (ZG-200L, Dongguan Zhenggong Mechanical and Electrical Equipment Technology Co., Ltd, Dongguan City, China) for 2 min. Then, zinc oxide (5 phr), stearic acid (2 phr), P-Silica/FS-SiO_2_ (30 phr), antioxidant 2246 (2 phr), accelerator CZ (1.5 phr), accelerator DM (0.5 phr), and sulfur (1.6 phr) were added sequentially to the masticated SBR over 30 min until a homogeneous mixture was formed [29]. Subsequently, the SBR compounds were stored at room temperature for 16 h before the optimal cure time (Tc_90_) was reached at 160 °C. Finally, the SBR compound was vulcanized according to Tc_90_ by heating the material to 160 °C under a pressure of 10 MPa in a heat press vulcanizer. The obtained vulcanizates were marked as SBR-P-silica, SBR-FS-5.3, SBR-FS-6.3, SBR-FS-7.3, SBR-FS-8.3, and SBR-FS-9.3.

### 2.4. Characterization and Performance Tests

#### 2.4.1. Characterization of the Fillers

The pore structure of P-silica/FS-SiO_2_ was characterized by a nitrogen adsorption technique using a pore structure analyzer (JWGB Sci & Tech Ltd., Beijing, China, JW-BK222). The specific surface area was determined using the Brunauer–Emmett–Teller (BET) equation, and the pore size distribution was calculated using the Barrett–Joyner–Halenda (BJH) model. The particle size distributions were examined using a laser particle sizer (Winner 2000E, Jinan Winner Particle Instruments Stock Co., Ltd. Jinan City, China). Scanning electron microscopy (SEM) images were captured by an electron microscope (Hitachi Ltd.,Tokyo, Japan, TM4000). A JEOL JEM-2100F (JEOL Japan Electronics Co., Ltd, Tokyo, Japan) transmission electron microscope (TEM) was used for the particle size and structure analysis of P-silica/FS-SiO_2_.

#### 2.4.2. Testing of the Filled SBR Composites

The curing characteristics of the SBR composites were obtained after 30 min of testing at 160 °C using a rheometer (MDR2000, Alpha Pro Tech, Ltd, Los Angeles, CA, USA). The scorch time (Tc_10_), optimum cure time (Tc_90_), minimum torque (ML), and maximum torque (MH) were determined. The mechanical properties of the rubber composites were determined following the national testing standards of China (GB/T528-2009 and GB/T529-2008), using a Universal Materials Testing Machine (model CSS-1102C, Changchun Testing Machine Research Institute, Changchun, City). When measuring the tensile strength and tear strength, the movement speed of the clamp was 500 mm/min. The dynamic storage modulus of the non-vulcanized SBR compound was obtained using the Rubber Processing Analyzer RPA2000 (Alpha Pro Tech, Ltd, Los Angeles, CA, USA), and the strain sweep test, with a strain range of 0.2% to 400%, was performed at a constant 1 Hz frequency and at 60 °C. A dynamic thermomechanical analysis (DMA) of the SBR composites was performed using a DMAQ800V system (TA Instruments, Newcastle, DE, USA). Testing was performed in the tensile mode with a dynamic strain of 0.25% and frequency of 10 Hz over the temperature range of −80 to 80 °C at a heating rate of 5 °C/min. The fracture morphology of the SBR vulcanizates was investigated by scanning electronic microscopy (Hitachi Ltd., Tokyo, Japan, TM4000).

#### 2.4.3. Crosslink Density of the SBR Composites

The dimensions of prepared vulcanized were 10 mm × 10 mm × 2 mm. The samples were weighed before being placed in a container with toluene. After swelling in the toluene for 72 h, the samples were taken and weighed immediately. To ensure the complete drying of the vulcanizate, the samples were dried in an oven at 70 °C for 48 h until the weight of the samples was constant. The crosslink density of the vulcanizates were calculated according to the Flory–Rehner [30] Equations (1)–(3).

(1) $$C_{d}=-\left[\ln\left(1-V_{r}\right)+V_{r}+\chi V_{r}^{2}]/[V_{s}\left(V_{r}^{\frac{1}{3}}-\frac{V_{r}}{2}\right)\right]$$

(2) $$V_{r}=\frac{V_{rubber}}{V_{rubber}+V_{solvent}}$$

(3) $$V_{r}=\left(\frac{m_{3}-m_{1}\times W_{f}}{\rho_{rubber}}\right)÷\left[\frac{m_{2}-m_{3}}{\rho_{solvent}}+\left(\frac{m_{3}-m_{1}\times W_{f}}{\rho_{rubber}}\right)\right]$$

where *C_d_* is the crosslinking density (mol/cm^3^), *V_r_* is the volume fraction of the rubber in the swollen gel, *V_s_* is the molar volume of toluene (106.87 cm^3^/mol), and χ is the SBR–toluene interaction parameter (here, χ = 0.41) [21]. In Equation (3), *m*_1_, *m*_2_, and *m*_3_ are the weights of the sample: *m_1_* is measured before swelling, *m*_2_ is measured after swelling, and *m_3_* is measured after drying. *W_f_* is the mass fraction of non-rubber components in the SBR vulcanizate. According to the formulation of the SBR composites described in Section 2.3, *W_f_* was 0.299. Finally, *ρ_rubber_* and *ρ_solvent_* are the densities of SBR-1502 (1.04 g/cm^3^) and toluene (0.867 g/cm^3^).

## 3. Results and Discussion

### 3.1. Characteristics of the Composite Filler Morphology of the Fillers

**Figure 1:**
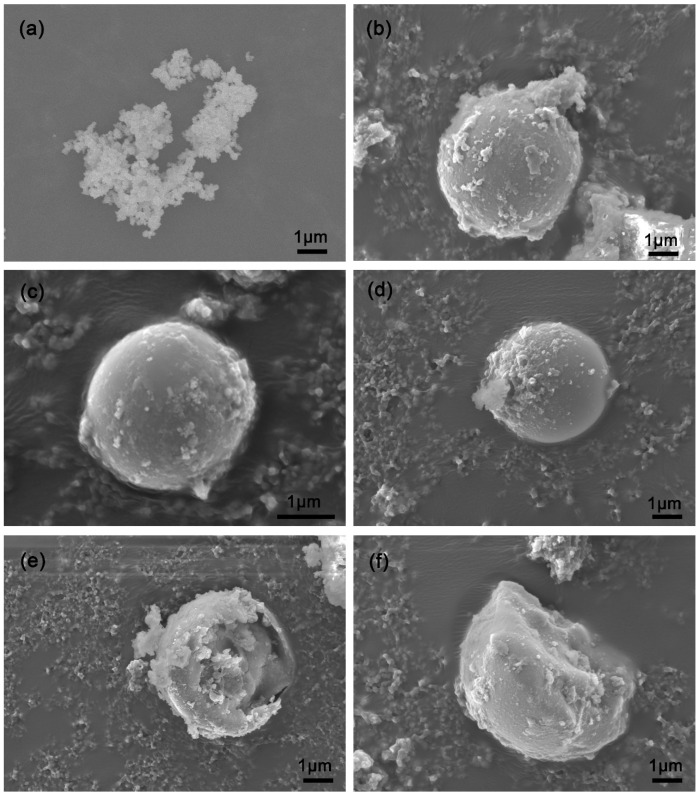
SEM images of (**a**) P-silica, (**b**) FS-5.3, (**c**) FS-6.3, (**d**) FS-7.3, (**e**) FS-8.3, (**f**) FS-9.3.

The morphological images of the P-silica and FS-SiO_2_ were captured by a scanning electron microscope (SEM). Figure 1a,b shows that the P-silica had a branch chain morphology comprised of smooth silica particle aggregates, whereas FS-SiO_2_ consisted of regular spheres with a rough surface. The spheres of FS-SiO_2_ became more irregular with the increase in the pH value of fillers (from 5.3–9.3). This was because the added sodium hydroxide solution inevitably reacted with the silica, and some internal pore structures collapsed, resulting in the random half-sphere morphology [31,32]. The spherical agglomerates of P-silica had a particle size in the range of tens of nanometers, while that of FS-SiO_2_ was less than 6 microns. The dispersion of FS-SiO_2_ exceeded that of P-Silica, although FS-SiO_2_ had a larger particle size than the primary particles of P-Silica, suggesting that a better rubber–filler interaction may exist between FS-SiO_2_ and the SBR matrix.

**Figure 2:**
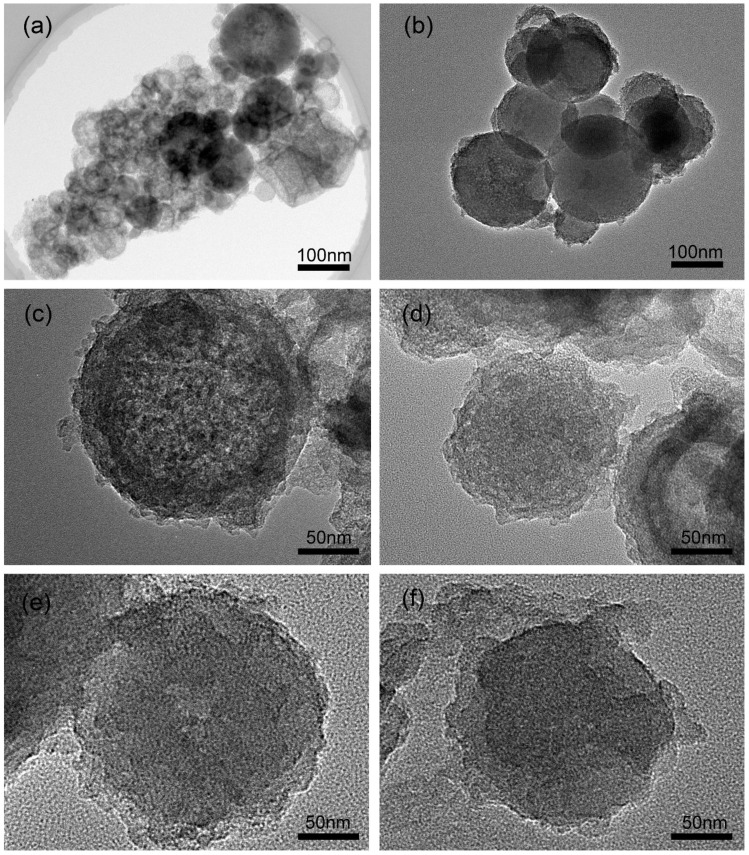
TEM images of (**a**) P-silica, (**b**) FS-5.3, (**c**) FS-6.3, (**d**) FS-7.3, (**e**) FS-8.3, (**f**) FS-9.3.

To further reveal the structural distinction between p-silica and FS-SiO_2_, transmission electron microscopy (TEM) was applied to observe the internal structure of these fillers. The P-silica is shown in Figure 2a. In order to observe the internal pore structures of FS-SiO_2_ more clearly through TEM, the spheres with particle sizes of less than 1 μm in each FS-SiO_2_ filler were used, as shown in Figure 2b–f. It can be seen that the FS-SiO_2_ showed a well-developed porous surface caused by the leaching of metal oxides, similar to Liu’s reported research [9]. Due to the random wedge-shaped surface channels, the interaction between the hydrochloric acid solution and the internal oxide of the sphere could be carried out, leading to a porous internal structure. The dendritic aggregates of P-silica consisted of several solid silica spheres with a smooth surface, as can be seen in Figure 2a, which had a higher degree of structure and weaker friction with the polymer matrix than that of FS-SiO_2_, as seen in Figure 2b–f. In addition, from the TEM images of FS-SiO_2_, we can also observe that the higher pH of the FS-SiO_2_ is, the more blurred the porous structure on the filler surface will be. This is because when the sodium hydroxide solution reacted with hydrogen ions hydrolyzed by the silicon hydroxyl group, the mesoporous structure inevitably collapsed with the dissolution of the silica.

### 3.2. Pore Structure of the Fillers

**Figure 3:**
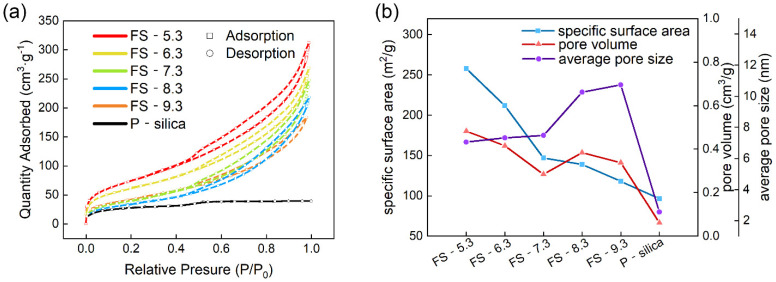
(**a**) N_2_ adsorption/desorption isotherms of P-Silica and FS-SiO_2_. (**b**) Specific surface area, pore volume, and average pore size of P-silica and FS-SiO_2_.

The adsorption/desorption isotherms of the FS-SiO_2_ samples prepared with different pH values are shown in Figure 3a. Table 1 shows the specific values of the the pore properties of P-silica and FS-SiO_2_. The specific surface area, the pore volume, and the average pore size of the P-silica and FS-SiO_2_ samples with different pH are shown in Figure 3b. The adsorption isotherms exhibited as type Ⅳ, as shown in Figure 3a, with type D hysteresis loops, which indicated that the form of the mesopores in FS-SiO_2_ was narrow and slit-like [28]. However, almost no pore structure existed in the P-silica, which was demonstrated by its type Ⅰ adsorption isotherm and small pore volume. It can be seen from Figure 3b that an increase in the pH of the FS-SiO_2_ led to a decrease in the specific surface area and pore volume, while the average pore size became larger. This is because the added sodium hydroxide solution inevitably reacted with the silica, and some micropores collapsed, resulting in the reduction in the specific surface area and the expansion of average pore size [31,32]. Obviously, P-silica had the lowest specific surface area, pore volume, and average pore size due to the lack of a pore structure. By contrast, the abundant internal and external pore structures of FS-SiO_2_ led to a large specific surface area, which meant that more active positions could make contact with the rubber matrix [33]. The physical entanglement of the molecular chain of the SBR matrix and the mesoporous structure of FS-SiO_2_ significantly affected the filler’s reinforcement ability. Thus, FS-SiO_2_ might serve as a good reinforcement filler.

### 3.3. Surface Silanol Groups

**Figure 4:**
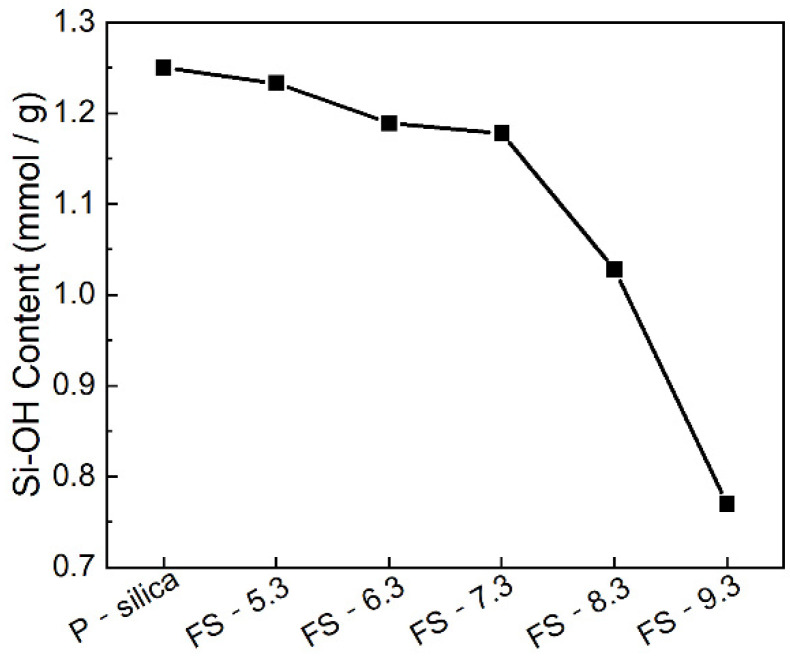
Si-OH content of P-silica and FS-SiO_2_.

As a kind of rubber filler, the silica’s reinforcement performance was determined by the amount of acidic hydroxyl on its surface [34]. However, excessive surface hydroxyl absorbed the accelerators and reduced the speed and degree of vulcanization. In addition, silica particles agglomerated owing to the hydrogen bond caused by the hydroxyl, which deteriorated its dispersion in the rubber matrix. Therefore, the study of the surface silanol groups of FS-SiO_2_ and P-silica helped us to fully understand how the amount of surface hydroxyl affects a filler’s reinforcement properties. In L.T. Zhuravlev’s [35] review article, surface silanol groups of amorphous silicas were divided into four types: (1) isolated silanol groups, ≡SiOH; (2) geminal silanol groups, =Si(OH)_2_; (3) vicinal silanol groups, or OH groups, connected through the hydrogen bond; and (4) internal silanol groups (not discussed). After burning at 600 °C in the preparation process of FS-SiO_2_, the remaining types of silanol groups were isolated, which included silanol and geminal silanol, according to Liu’s research [9]. Figure 4 illustrates the Si-OH contents of P-silica and FS-SiO_2_ and shows that a decrease in the Si-OH content led to an increase in the pH of FS-SiO_2_, which was caused by the hydrolysis reaction of Si-OH, promoted by sodium hydroxide. This phenomenon indicated that the amount of surface hydroxyl was associated with the addition of sodium hydroxide.

### 3.4. Particle size of the Fillers

Analyzing the particle size of a filler is of great significance for improving its reinforcement performance, especially in the case of a small particle filler. The particle size cumulative volume frequencies (D_10_, D_50_, and D_90_) and proportion of particles sized smaller than 5 μm and 10 μm are shown in Table 2. The particle size of FS-SiO_2_ was found to be significantly smaller than that of the P-silica. FS-6.3 was discovered to have the smallest D_av_ and D_90_ of all the samples (3.606 μm, 6.475 μm), with 76.633% of particles less than 5 μm, indicating a narrow and uniform particle size distribution. In addition, P-silica had the widest particle size distribution and the largest average particle size, with the D_10_, D_90_, and D_av_ values being 3.326 μm, 12.263 μm, and 6.706 μm, respectively, which were larger than the corresponding values for the other five samples. Additionally, only 37.066% of the P-silica particles were smaller than 5 μm, from which we can infer that the reinforcement property of P-silica might be worse than that of FS-SiO_2_. The decreasing tendency of the D_90_ and D_av_ of FS-SiO_2_ with different pH values validated the observation that adding sodium hydroxide solutions is conducive to reducing the particle size.

### 3.5. Cure Characteristics

Table 3 summarizes the cure characteristics of the P-silica- and FS-SiO_2_-filled vulcanizates. T_s1_ is the time for the onset of the cure, Tc_90_ is the optimum vulcanization time, and Tc_10_ is the scorch time, related to the processing safety. Both Ts_1_ and Tc_90_ decreased with the increasing pH of FS-SiO_2_. On the one hand, due to the reaction between the sodium hydroxide and silica, the FS-SiO_2_ with a high pH lost part of the mesoporous structures, and the accelerator adsorbed by the filler decreased, which sped up the vulcanization of the rubber compound. On the other hand, after the treatment of the NaOH solution, the number of silanol groups on the surface of FS-SiO_2_ gradually decreased, and the number of sites where the active vulcanizing agent bound to the α-H of the rubber molecular chain increased, which also reduced the T_s1_ and Tc_90_. Although the pore structure of FS-SiO_2_ restricted the movement of the rubber molecular chains, there still existed some residual air in the pores, which led to a lower thermal conductivity [36,37]. This is why the FS-SiO_2_-filled rubber took a longer time to cure than the P-silica-filled rubber. In addition, the scorch time of the FS-SiO_2_-filled compounds was almost indistinguishable from the commercial P-silica-filled compounds.

ML reflected the fluidity of the compound during processing, and compounds with lower MLs had better processing properties. Compared with P-silica, the dispersed and regular spherical structure of FS- SiO_2_ endowed its filled compound with better fluidity. Obviously, the latter compound had a better processing performance. The data on the ML are shown in Table 3. A comparison of the SEM images of the morphologies of FS-SiO_2_ and P-silica proves that the crosslink density can be characterized to some extent by the Δ torque (MH-ML). The P-silica with smaller primary particles and a higher degree of structure formed more bonded rubber [38]. Fewer accelerators were adsorbed by P-silica owing to its lack of pore structures. However, the particles of P-silica aggregated more easily, which degraded the crosslink density of the vulcanized rubber. With the increase in the FS-SiO_2_ pH, the adsorbed accelerator decreased due to the reduction in the pore structure, and the crosslinking density of the vulcanizate gradually increased. However, the excessive reduction in the number of pore structures weakened the ability of the filler to confine the segmental motion of rubber molecular chain, leading to a decrease in the rubber–filler interaction.

### 3.6. Mechanical Properties

**Figure 5:**
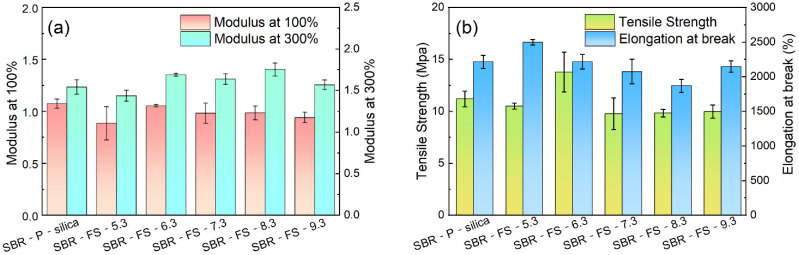
Mechanical properties of the SBR rubber filled with P-silica and FS-SiO_2_: (**a**) modulus at 100% and 300%, (**b**) tensile strength and elongation at break.

The effects of the different fillers on the mechanical properties of the SBR composites are shown in Figure 5. The specific values of mechanical properties of the SBR rubber composites with P-silica and FS-SiO_2_ are tabulated in Table 4. Figure 5a shows that FS-SiO_2_ with a pH no less than 6.3 imparted a higher tensile modulus than the P-silica-filled vulcanizates at 300% strain. As shown in Figure 5b, SBR-FS-5.3 had the highest elongation at break, and SBR-FS-6.3 had the best tensile strength, exceeding that of the SBR-P-silica composite. This was because the segmental motion of the rubber chains was confined within the mesoporous channels [39]. Both the elongation at break and tensile strength of the SBR composites decreased with the increase in the pH of FS-SiO_2_. The N_2_ adsorption and desorption tests and the SEM morphology of the tensile fracture surface of the SBR composites confirmed that the filler with a higher pH value had a smaller specific surface area and a more irregular spherical structure. However, fillers with smaller specific surface areas adsorbed fewer rubber molecular chains, and the decrease in the tensile strength and elongation at break of the SBR composites was attributed to weaker rubber–filler interactions. Overall, FS-6.3 achieved a balance between the adsorption of accelerators and the promotion of rubber–filler interactions.

According to the hypothesis of interfacial slip [40], when the rubber was under tension, the polymer chains slipped on the surface of the filler to form an orientation. Compared with the branch chain formed of silica aggregates, the independent and regular spherical structure of FS-SiO_2_ was more likely to be pulled and rotated by the rubber molecular chain, leading to the easier formation of the orientation of the rubber molecular chain [41]. Moreover, the friction between the rubber molecular chain and spherical FS-SiO_2_ offset part of the external force [42]. It should be noted that the frictional heat generated during sliding was critical for releasing the strain energy, which helped to prevent the failure and fracture of the polymer matrix. Hence, the elongation at break and tensile strength of SBR-FS-5.3/ 6.3 improved. However, with the increase in the pH of fillers, the specific surface area and the degree of the regular spherical structure of FS-SiO_2_ reduced, and the elongation at break and tensile strength of the compound were weakened.

**Figure 6:**
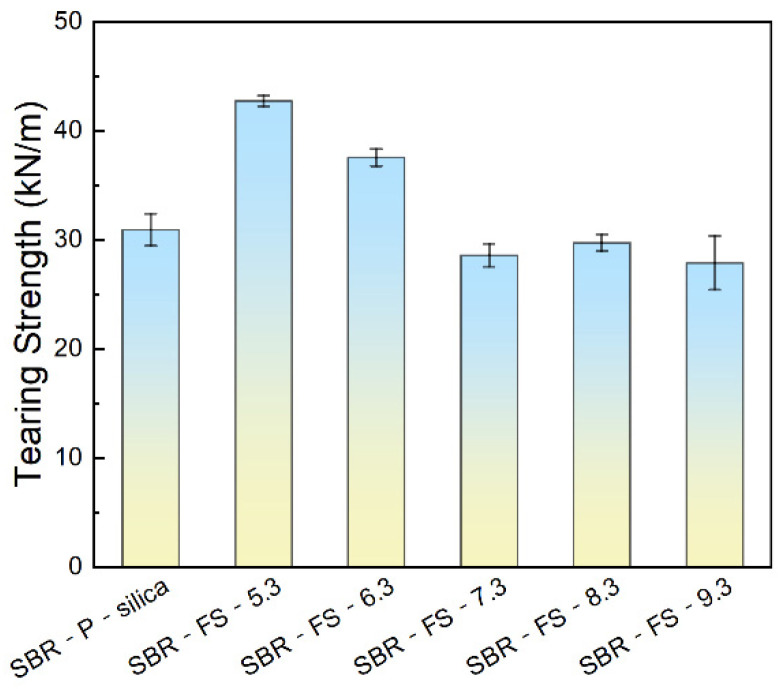
Tearing strength of the SBR rubber filled with P-silica and FS-SiO_2_.

Figure 6 presents the change in the tearing strengths of the SBR composites. The tearing strengths of SBR-FS-5.3 and SBR-FS-6.3 exceeded that of SBR-P-silica. The micro-crack propagation was obstructed by the monodispersed particles of FS-SiO_2_. The SBR-FS-6.3 composite performed with a better tensile strength and tear strength than the SBR-P-silica composite and had the same level of elongation at break. It has great potential to be used as a substitute for precipitated silica.

### 3.7. Crosslink Density of SBR Composites

**Figure 7:**
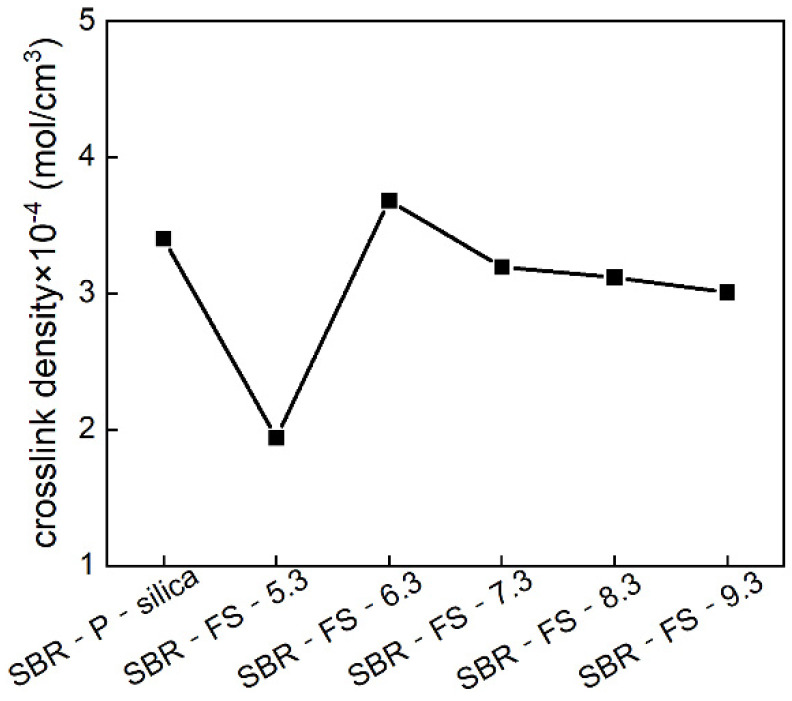
Crosslink density of the SBR rubber filled with P-silica and FS-SiO_2_.

Figure 7 illustrates the crosslink density of the vulcanizates filled with P-silica and FS-SiO_2_. The crosslink density was measured by the swelling test based on the Flory–Rehner equation [30]. In general, a higher crosslink density indicates a better mechanical property [38]. Furthermore, strong filler–rubber interactions provide excess crosslinking points, thus leading to a higher crosslink density. In Figure 7, the crosslink density increased rapidly as the pH of the filler reached 6.3 and then dropped slightly. This is because the FS-6.3 had the most appropriate amount of surface hydroxyl and a large number of mesoporous channels, which caused the highest crosslink density [39].

In short, FS-SiO_2_, with a high specific surface area, an appropriate amount of surface hydroxyl, and a small particle size, contributed to a higher crosslink density, which enhanced the mechanical performance.

### 3.8. Microstructure of SBR Composites

**Figure 8:**
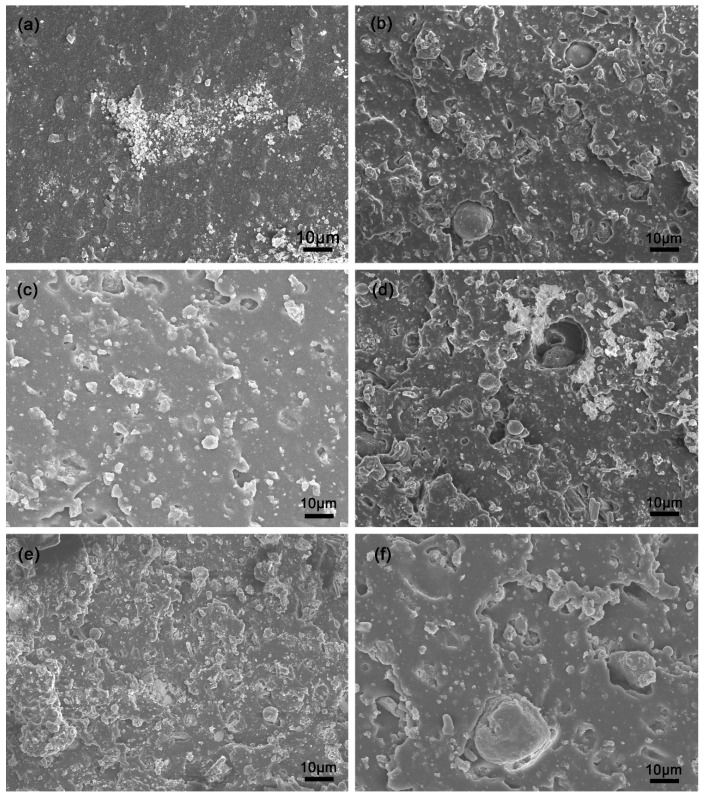
SEM showing the tensile fracture surfaces of the SBR composites filled with P-silica and FS-SiO_2_, (**a**) SBR-P-Silica, (**b**) SBR-FS-5.3, (**c**) SBR-FS-6.3, (**d**) SBR-FS-7.3, (**e**) SBR-FS-8.3, (**f**) SBR-FS-9.3.

The dispersion morphology of the filler in the rubber matrix and the adhesion performance between the matrix and filler play important roles in the properties of SBR composites [18,43]. The fracture morphologies of the vulcanizates filled with P-silica and FS-SiO_2_ are shown in Figure 8. The fracture morphology of the composite filled with P-silica can be seen in Figure 8a. The fracture surfaces look smooth and the P-silica appears significantly aggregated, indicating the poor dispersion of P-silica particles. On the other hand, the FS-SiO_2_ particles were dispersed more uniformly in the SBR matrix, as shown in Figure 8b–f. Compared to SBR-P-silica, the fracture surfaces of SBR-FS-SiO_2_ were quite rough, and many dispersed spherical particles were embedded in the rubber matrix. However, in Figure 8b,d, cavities can be observed on the boundaries between the spherical FS-SiO_2_ and the rubber matrix due to the excessive amount of surface hydroxyl and unsuitable particle size. It is noted that the contact boundary between FS-6.3 and the SBR matrix was very indistinct, and the SBR could easily wet the FS particle surfaces, indicating a better surface adhesion between these two phases. Additionally, very few large particles can be observed in SBR-FS-6.3, which is one of the reasons explaining why SBR-FS-6.3 had the best tensile strength. Figure 8d–f illustrates the fracture morphologies of the vulcanizates filled with FS-7.3, FS-8.3, and FS-9.3. The spherical structure of FS-SiO_2_ became more irregular, resulting in worse mechanical properties [44]. This might be due to the cutting effect of the irregular filler on the rubber molecular chain [24]. Therefore, FS-6.3 dispersed uniformly and had a good compatibility with the SBR matrix, which resulted in the strong reinforcement of the SBR.

### 3.9. Dynamic Mechanical Analysis (DMA)

**Figure 9:**
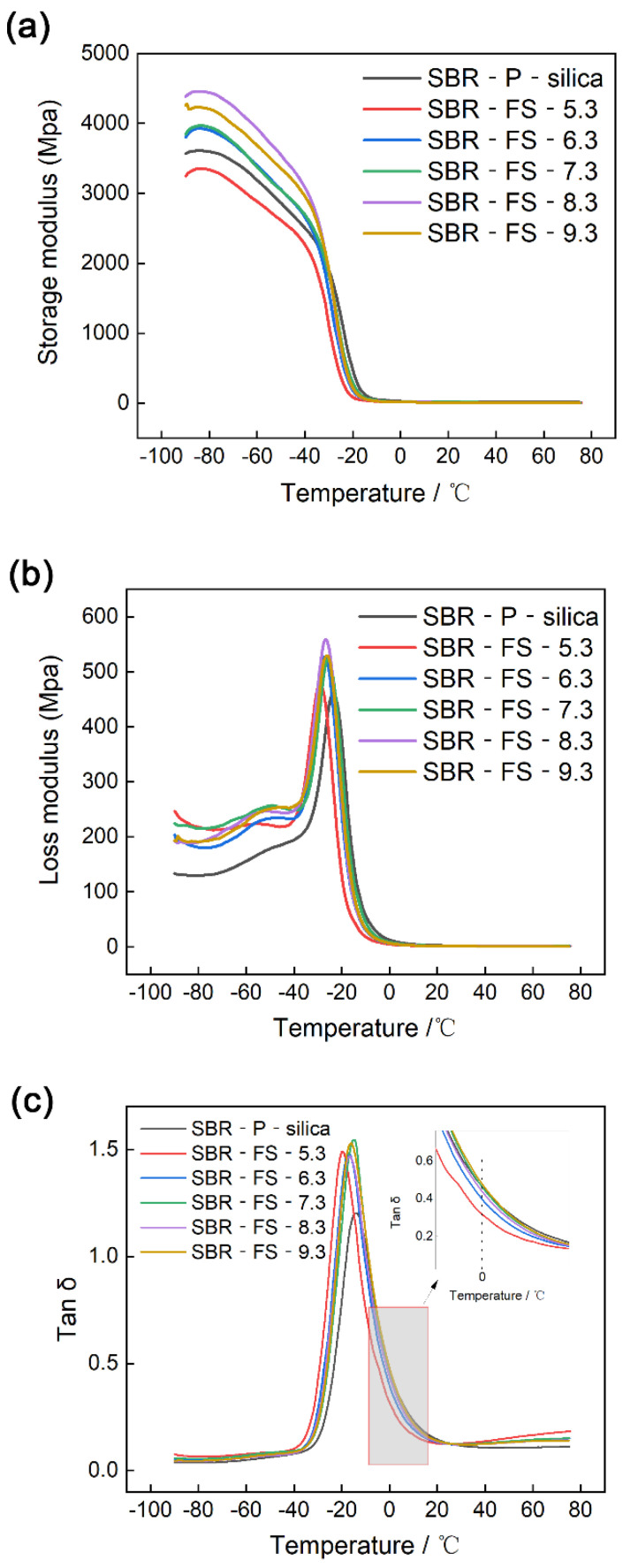
Storage modulus (**a**), loss modulus (**b**), and tan δ (**c**) as functions of temperature of the SBR vulcanizates filled with P-silica and FS-SiO_2_ (the insert is a magnification of the temperature, around 0 °C).

Generally, rubber undergoes three physical states with increasing temperature, which are the glassy, glass transition, and rubbery states, respectively. Dynamic mechanical analysis (DMA) technology allows researchers to investigate the filler–rubber interactions and molecular mobility based on the storage/loss modulus and tan δ of composites at different temperatures [45]. The storage/loss modulus and tan δ, as functions of the temperature of SBR-P-silica/SBR-FS-SiO_2_, are shown in Figure 9. It is illustrated in Figure 9 that the storage modulus declined drastically at around −30 °C, which apparently demonstrated that the glass transition had occurred. This can be explained by the immobility of the molecular chains at a low temperature, which, in contrast, can move easily at a high temperature. It was clearly seen that the composite SBR-FS-8.3 exhibited the highest storage modulus in the glassy region, as shown in Figure 9a, which might be attributed to the fact that FS-8.3 had the largest number of particles of a size below 10 μm (Table 2). On the other hand, the composite SBR-FS-5.3 showed the lowest storage modulus. Not only the sphere structure but also the high content of surface hydroxyl of FS-5.3 in the FS-SiO_2_ fillers led to the free void volume between the surface of the filler and rubber matrix. Figure 9b shows the values of the loss modulus of the composites. It can be seen that SBR-P-silica had the lowest maximum heat dissipation, while SBR-FS-8.3 had the highest value. This might be attributed to the fact that the motion of the rubber segments was confined more effectively by P-silica than FS-SiO_2_ in the glass transition region [46].

Figure 9c illustrates the variation in the loss factor (tan δ) as a function of temperature of the P-silica-filled and the FS-SiO_2_-filled SBR vulcanizates with different pH values. Generally, tan δ relates to the damping performance of a rubber composite [47], which is primarily determined by the nature of the rubber matrix, reinforcement filler, and their interface. Furthermore, interfacial delamination and slippage on the surface between the filler and matrix exercise considerable influences on the damping properties. The appearance of a damping peak is associated with the migration of side groups and molecular chains in the glassy transition regions. Hence, a higher maximum tan δ_max_ demonstrates a better molecular mobility. It can be seen from Figure 9c that the FS-SiO_2_-filled vulcanizate showed a higher tan δ_max_ than that of the P-silica-filled vulcanizate, which indicated that it had superior damping properties and elastomeric characteristics. Therefore, composites filled with FS-SiO_2_ have the potential to be used as shock absorption and noise reduction materials [48,49]. This might be due to the fact that spherical FS-SiO_2_ particles roll and rub against the rubber chain segment more easily, resulting in a higher energy dissipation. Additionally, the temperature at the loss factor peak center represented the estimated glassy transition temperature (Tg). It was found that SBR-P-silica showed the highest Tg, and with the increase in the FS-SiO_2_ pH, the Tg became higher. The degree of restriction of the chain segment movement by the filler is the main influencing factor. Furthermore, the tan δ of SBR-FS-9.3 was higher than that of SBR-P-Silica at 0 °C owing to its irregular spherical structure, which led to a better wet skid resistance [50,51].

### 3.10. Rubber Processing Analysis (RPA)

**Figure 10:**
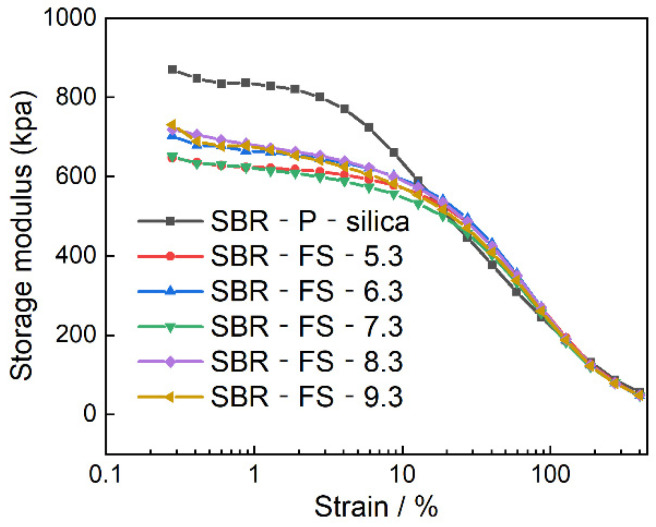
RPA strain sweep of SBR composites filled with P-silica and FS-SiO_2_.

Generally, the strength of the filler network can be described by the Payne effect, which indirectly reflects the dispersion of the fillers [52,53]. Figure 10 shows that the storage modulus (G’) of the non-vulcanized SBR composites decreased nonlinearly as the strain increased, which is known as the Payne effect [54]. In addition, the composites exhibited a higher G’ in the low-strain region due to the filler network, which caused strong filler–filler interactions [55]. As the strain started to increase, the filler networks began to be destroyed but then rebuilt quickly. When the strain increased by more than 10%, the filler networks were gradually ruined, as reflected by the sharply decreasing tendency of G’. Finally, the filler networks were destroyed permanently as the strain increase to about 400%, and the G’ of the SBR composites decreased to the same level. Therefore, ΔG’ represented the strength of the filler networks in the filled rubber compounds. It is seen from Figure 10 that SBR-P-silica yielded the highest value of ΔG’, indicating the strongest filler–filler interactions in the SBR-P-silica composite. On the other hand, the SBR composites filled with FS-SiO_2_ showed lower ΔG’ than the SBR-P-silica, which demonstrated a weaker Payne effect and a more uniform filler dispersion. This might be due to the single spherical structure and lower amount of surface hydroxyl, reducing the formation of filler networks and revealing weaker filler–filler interactions and stronger filler–rubber interactions established by FS-SiO_2_. Table 5 summarizes the values of ΔG’ of the SBR composites, and it is noted that the range of the filler’s pH, being 5.3–7.3, led to a uniform dispersion.

## 4. Conclusions

Acid leaching, calcination, and pH adjustment were used to modify coal gasification fine slag, increase the specific surface area, and decrease the amount of surface hydroxyl. Although P-silica and FS-SiO_2_ are both silica, their microstructures differ greatly. P-silica aggregated to create a chain branch structure, while FS-SiO_2_ existed as isolate spheres. Therefore, the FS-SiO_2_ in the SBR matrix showed a weaker Payne effect than P-silica, which indicated that FS-SiO_2_ dispersed more uniformly than P-silica in the rubber matrix. The tensile strength of SBR-FS-6.3 was superior to SBR-P-Silica, and the tearing strengths of SBR-FS-5.3 and SBR-FS-6.3 were better than that of the vulcanizate filled with P-silica.

The FS-SiO_2_ with a pH of 6.3 showed the highest degree of crosslinking and the best reinforcement properties among the SBR composites, offering a tensile strength of 13.78 Mpa owing to its excellent compatibility with the SBR matrix and uniform dispersion. The tearing strength values of SBR-FS-5.3 and SBR-FS-6.3 reached 42 KN/m and 37 KN/m, respectively, which were better than that of SBR-P-silica. FS-SiO_2_, as a filler, reduces the filler–filler interactions while enhancing the filler–rubber interactions, resulting in a higher wet skid resistance of the vulcanizates. The adjustment of the pH value of the FS-SiO_2_ resulted in the reduction in the curing time (Tc_90_). These results indicated that the FS-SiO_2_ prepared by the new method performed better in accelerating the vulcanization and reinforcing the rubber. Consequently, CFS can replace commercial precipitated silica as a reinforcement filler, which can reduce the production costs, as well as environmental pollution.

## Figures and Tables

**Table 1:** The pore properties of P-silica and FS-SiO_2_.

Samples	Specific Surface Area (m^2^/g)	Pore Volume (cm^3^/g)	Average Pore Size (nm)
FS-5.3	258	0.483	7.054
FS-6.3	212	0.415	7.320
FS-7.3	147	0.284	7.484
FS-8.3	139	0.384	10.261
FS-9.3	118	0.338	10.738
P-silica	96	0.061	2.557

**Table 2:** Particle size characteristics (D_10_, D_50_, D_90_, and D_av_) and the proportion of particles sized less than 5 μm and 10 μm of P-silica and FS-SiO_2_.

Sample	D_10_ (μm)	D_50_ (μm)	D_90_ (μm)	D_av_ (μm)	Standard Deviations	<5 μm	<10 μm
FS-5.3	1.173	4.583	9.811	5.501	5.490	56.844%	90.180%
FS-6.3	1.265	3.258	6.475	3.606	2.332	76.633%	98.840%
FS-7.3	1.152	3.855	6.536	3.809	1.663	69.030%	100.000%
FS-8.3	1.457	4.247	6.758	4.168	2.170	64.174%	99.928%
FS-9.3	1.268	4.048	6.745	4.021	2.293	66.585%	99.686%
P-silica	3.326	5.696	12.263	6.706	4.012	37.066%	84.244%

**Table 3:** Cure characteristics of rubber composites filled with P-silica and FS-SiO_2_ (T_c10_, T_c90_, t_s1_, ML, MH, MH-ML).

Sample	T_c10_ (min)	T_c90_ (min)	t_s1_ (min)	ML (dNm)	MH (dNm)	MH-ML (dNm)
SBR-P-silica	3.83	19.41	3.84	2.32	11.14	8.82
SBR-FS-5.3	4.35	24.41	5.58	2.19	8.28	6.09
SBR-FS-6.3	3.49	22.96	3.99	2.28	12.28	10.00
SBR-FS-7.3	3.58	22.24	4.06	1.94	9.92	7.98
SBR-FS-8.3	3.14	21.37	3.85	2.16	9.85	7.69
SBR-FS-9.3	3.55	20.54	3.78	2.03	10.68	8.65

**Table 4:** Mechanical properties of the SBR rubber composites with P-silica and FS-SiO_2_.

Sample	Modulus at 100%	Modulus at 300%	Tensile Strength (Mpa)	Elongation at Break (%)	Tearing Strength (kN/m)
SBR-P-silica	1.08 ± 0.04	1.54 ± 0.09	11.19 ± 0.77	2215.2 ± 94.3	30.93 ± 1.46
SBR-FS-5.3	0.89 ± 0.16	1.44 ± 0.07	10.50 ± 0.29	2494.7 ± 38.1	42.74 ± 0.49
SBR-FS-6.3	1.05 ± 0.01	1.69 ± 0.02	13.78 ± 1.91	2215.0 ± 105.0	37.55 ± 0.77
SBR-FS-7.3	0.98 ± 0.09	1.64 ± 0.06	9.76 ± 1.53	2072.8 ± 177.2	28.59 ± 1.06
SBR-FS-8.3	0.99 ± 0.06	1.75 ± 0.08	9.83 ± 0.36	1869.0 ± 93.9	29.74 ± 0.77
SBR-FS-9.3	0.94 ± 0.05	1.57 ± 0.06	9.98 ± 0.64	2146.4 ± 81.3	27.90 ± 2.47

**Table 5:** The delta storage modulus (ΔG’) of SBR composites filled with P-silica and FS-SiO_2_.

	SBR-P-silica	SBR-FS-5.3	SBR-FS-6.3	SBR-FS-7.3	SBR-FS-8.3	SBR-FS-9.3
ΔG’	814.55	600.15	655.81	602.83	670.18	683.12

## Data Availability

The authors declare that all data used are available upon request.
